# Genome-wide linkage analysis and regional fine mapping identified variants in the *RYR3* gene as a novel quantitative trait locus for circulating adiponectin in Chinese population

**DOI:** 10.1097/MD.0000000000005174

**Published:** 2016-11-04

**Authors:** Yi-Cheng Chang, Yen-Feng Chiu, Chih-Tsueng He, Wayne Huey-Herng Sheu, Ming-Wei Lin, Todd B. Seto, Themistocles Assimes, Yuh-Shan Jou, Lynn Su, Wei-Jei Lee, Po-Chu Lee, Shu-Huei Tsai, Lee-Ming Chuang

**Affiliations:** a Graduate Institute of Medical Genomics and Proteomics, National Taiwan University; b Department of Internal Medicine, National Taiwan University Hospital; c Institute of Biomedical Science, Academia Sinica, Taipei; d Division of Biostatistics and Bioinformatics, Institute of Population Health Sciences, National Health Research Institutes, Zhunan; e Department of Internal Medicine, Tri-Service General Hospital Songshan Branch, Taipei; f Division of Endocrinology and Metabolism, Department of Internal Medicine, Taichung Veterans General Hospital, Taichung; g Institute of Public Health, National Yang-Ming University, Taipei, Taiwan; h Center for Outcomes Research and Evaluation, Non-Invasive Cardiology Laboratory, The Queen’s Medical Center, Honolulu, HI; i Department of Medicine, Stanford University School of Medicine, Stanford, CA; j Graduate Institute of Molecular Medicine, National Taiwan University, Taipei; k Department of Surgery, Ming-Sheng General Hospital, Taoyuan; l Department of General Surgery, National Taiwan University Hospital; m Institute of Preventive Medicine, College of Public Health, National Taiwan University, Taipei, Taiwan.

**Keywords:** adiponectin, Chinese, genome-wide, *RYR3*, SAPPHIRe

## Abstract

Adiponectin is adipocyte-secreted cytokine with potent insulin-sensitizing action in peripheral tissues. The heritability of plasma adiponectin is high in Han Chinese population.

To identify genetic loci influencing plasma adiponectin levels in Chinese population, we performed a genome-wide linkage scan in 1949 Chinese participants of the Stanford Asia-Pacific Program for Hypertension and Insulin Resistance family study and mapped a quantitative trail locus located on chromosome 15 at 31 cM (logarithm of odds = 3.04) with 1-logarithm of odds support interval at 24 to 34 cM. Within this mapped region, we further genotyped a total of 68 single-nucleotide polymorphisms in 12 genes. Association analysis revealed that haplotypes composed of single-nucleotide polymorphisms in the *ryanodine receptor 3* (*RYR3*) gene had strongest association with plasma adiponectin. *RYR3* haplotypes were also associated with systolic (*P* = 0.001) and diastolic (*P* = 7.1 × 10^−4^) blood pressure and high-density lipoprotein cholesterol (*P* = 1.4 × 10^−4^). Furthermore, an inverse relationship between expression of *RYR3* and *adiponectin* was observed in human abdominal adipose tissue. In conclusion, a genome-wide linkage scan and regional association fine-mapping identified variants in the *RYR3* gene as a quantitative trail locus for plasma adiponectin levels in Chinese population.

## 1 Introduction

Adiponectin is an adipocyte-secreted protein with potent insulin-sensitizing action in peripheral tissues such as skeletal muscle and liver. Plasma adiponectin levels are negatively associated with plasma triglycerides, measures of obesity, insulin resistance, and risk of type 2 diabetes but are positively associated with plasma high-density lipoprotein cholesterol (HDL-C) levels.^[1–3]^ The heritability of plasma adiponectin level is high (30–70%) in European population.^[4,5]^ A large-scale multiethnic meta-analysis of genome-wide association analysis (GWAS) involving 45,891 individuals identified variants in the *LYPLAL1*, *GNL3*, *TSC22D2*, *ADIPOQ*, *VEGFA*, *TRIB1*, *PDE3A*, *GPR109A*, *DNAH10*, *CMIP*, *CDH13*, *ZNF664*, and *PEPD* genes associated with plasma adiponectin.^[6]^ A GWAS of participants in the Nurses’ Health Study confirmed the association of *ADIPOQ* variants and found a new locus in the *FER* gene associated with plasma adiponectin.^[7]^ Another meta-analysis of GWAS involving 14,733 Europeans also confirmed the association of *ADIPOQ* variants and revealed a novel locus in the *ARL15* gene associated with plasma adiponectin.^[9]^ Another GWAS in Filipino women identified a novel signal near *KNG1* gene.^[8]^ A recent meta-analysis of GWAS in 7827 East Asians confirmed the association of variants at *CDH13*, *ADIPOQ*, *PEPD*, *CMIP*, *ZNF664*, and *GPR109A* genes and further identified a novel variant at *WDR11-FGFR2* gene associated with plasma adiponectin.^[9]^ These variants are also associated with triglycerides, HDL-C, blood pressure, measures of obesity, type 2 diabetes, and cardiometabolic outcomes.^[6,7,10,11]^

We have previously demonstrated a high heritability (h^2^ = 0.64) of plasma adiponectin in Chinese population.^[12]^ However, no genetic association study for plasma adiponectin has been carried out in Han Chinese population. In a previous genome-wide linkage analysis for plasma adiponectin levels using 376 microsatellite markers in the Stanford Asia-Pacific Program for Hypertension and Insulin Resistance (SAPPHIRe) Chinese family cohort,^[12]^ we identified a single peak with logarithm of odds (LOD) of 3.19 at 39 cM of chromosome 15. In this study, we sought to refine this linkage signal using additional microsatellite markers and performed regional association fine mapping using 68 single-nucleotide polymorphism (SNP) markers. We identified genetic variants in the *ryanodine receptor 3* (*RYR3*) gene associated with plasma adiponectin in Han Chinese.

## 2 Methods

### 2.1 The SAPPHIRe study cohort

The SAPPHIRe was a collaborative study that was part of the Family Blood Pressure Program of the National Heart, Lung, and Blood Institute of the National Institutes of Health originally designed to investigate the genetic determinants of hypertension and insulin resistance in Chinese individuals. The study collected sibling pairs who were either concordant or discordant for high blood pressure. Detailed descriptions of the study cohort were published in our previous work.^[12,13]^ Hypertension was defined as systolic blood pressure ≧140 mm Hg, diastolic blood pressure ≧90 mm Hg, or use of medications for high blood pressure. Individuals with pre-existing chronic illness such as diabetes, cancer, or diseases of the heart, liver, or kidney were excluded. A total of 1153 subjects of Han Chinese descent from 392 families were enrolled at baseline. The institutional review board of Tri-Service General Hospital in Taiwan, the National Taiwan University Hospital (NTUH) Research Ethics Committee, the institutional review board of Taipei Veterans General Hospital, and the institutional review board of Taichung Veterans General Hospital approved this study. Written informed consent was obtained from each participant. The baseline characteristics of participants are summarized in Table 1.

**Table 1:**
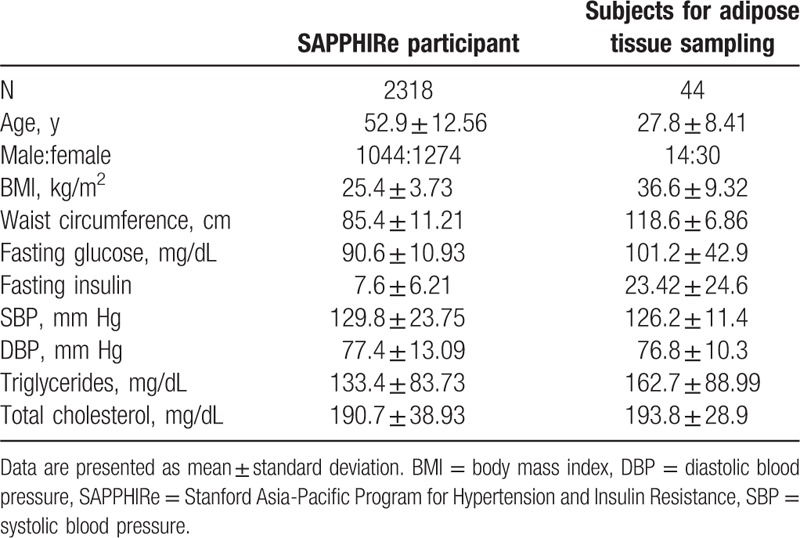
Characteristics of study participants.

### 2.2 Selection of tag SNPs and genotyping

Within the linkage region, we selected 68 SNPs in 12 genes including *cholinergic receptor, nicotinic, alpha 7* (*CHRNA7*, HGCN ID: 1960), *Rho GTPase activating protein 11A* (*ARHGAP11A*, HGCN ID: 15783), *secretogranin V* (*SCG5*, HGCN ID: 10816), *gremlin-1 precursor* (*GREM1*, HGCN ID: 2001), *RYR3* (HGCN ID: 10485), *cholinergic receptor, muscarinic 5* (*CHRM5*, HGCN ID: 1954), *solute carrier family 12, member 6* (*SLC12A6*, HGCN ID: 10914), *nuclear protein in testis* (*NUT*, HGCN ID: 29919), *PLSC domain containing protein* (*AYTL3*, HGCN ID: 30059), *golgin subfamily a, 8A* (*GOLGA8A*, HGCN ID: 31972), *aquarius* (*AQR*, HGCN ID: 29513), and *RAS guanyl releasing protein 1* (*RASGRP1*, HGCN ID:9878) genes from the HapMap Chinese Beijing database (HapMap genome browser release #24) (http://www.hapmap.org) based on physical intervals (Table 2).^[14]^ The average physical interval is 17.1 kb. The average call rate is 95.48%. The concordance rate of this system based on 160 genotyping duplications was 99.38%.

**Table 2:**
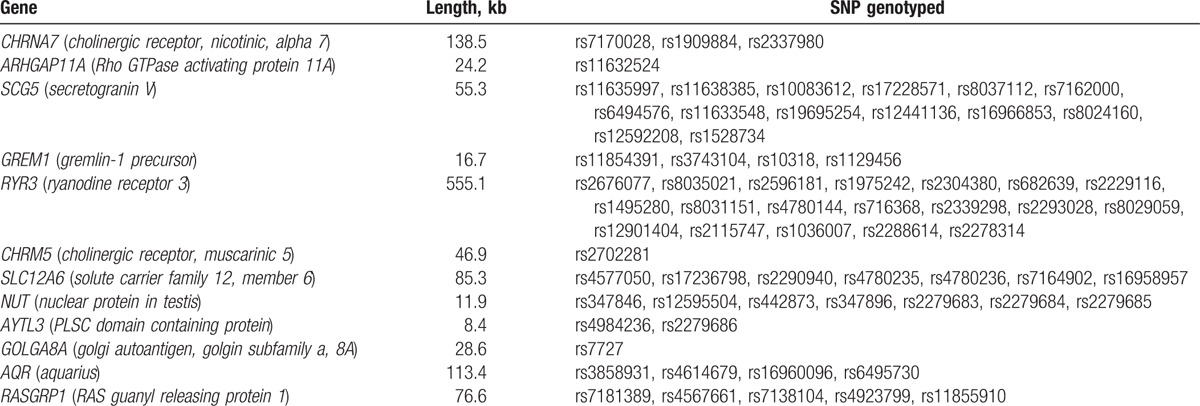
Single-nucleotide polymorphisms (SNPs) genotyped in genes within linkage region.

### 2.3 Subjects for measurement of gene expression in adipose tissue

We recruited 44 adult subjects undergoing bariatric surgery or elective abdominal surgery such as cholecystectomy or partial hepatectomy in Ming-Sheng General Hospital and Yunlin branch of NTUH in Taiwan. Abdominal omental adipose tissues were sampled in a fasting state during surgery and were placed in liquid nitrogen immediately until processing. The study was approved by the institutional review board of Ming-Sheng General Hospital and National Taiwan University Hospital. Written informed consent was obtained from each patient. Their baseline characteristics are summarized in Table 1.

### 2.4 Reverse transcription and quantitative real-time PCR

Total RNA was isolated using REzol C&T reagent (Protech, Taipei, Taiwan) and reverse transcribed with SuperScript III (Invitrogen, Carlsbad, CA) according to the manufacturer’s instructions. Polymerase chain reaction amplification was performed using LightCycler FastStart DNA Master Plus SYBR (Roche, Mannheim, Germany). Each sample was analyzed in duplicate and calibrated to the cyclophilin A housekeeping gene. The primer used for *RYR3* was PPH20011A (BD Bioscience, New Jersey, USA). The primers used for *PPLA* (cyclophilin A) were forward (5′-GCATACGGGTCCTGGCATCTTGTCC-3′) and reverse (5′-ATGGTGATCTTCTTGCTGGTCTTGC-3′).

### 2.5 Statistical analysis

The genome-wide scans were performed for logarithm-transformed adiponectin with adjustments for age and gender. Variance component model was used for linkage analysis using the Sequential Oligogenic Linkage Analysis Routines computer package.^[15]^ The detailed description of linkage analysis was published in our previous work.^[12,13]^ Likelihood ratio tests were used to test for the null hypothesis of no linkage. LOD scores were calculated as logarithm to base 10 of the likelihood ratios. A LOD score exceeding 3.3 was considered to be of genome-wide significance for evidence of linkage, whereas a LOD score >1.9 was suggestive of evidence for linkage. One-unit LOD support intervals were obtained by identifying the peak for the maximum LOD score on the plot of the linkage results, dropping down 1 LOD unit and finding the chromosomal region defined by the shoulders of the curve. For regional fine mapping, we used family-based association tests for single-SNP and haplotype association analysis.^[16]^ Hardy–Weinberg equilibrium test was performed before marker–trait analysis.

## 3 Results

### 3.1 Fine mapping of genome-wide linkage signal identified *RYR3* as QTL for plasma adiponectin

**Figure 1:**
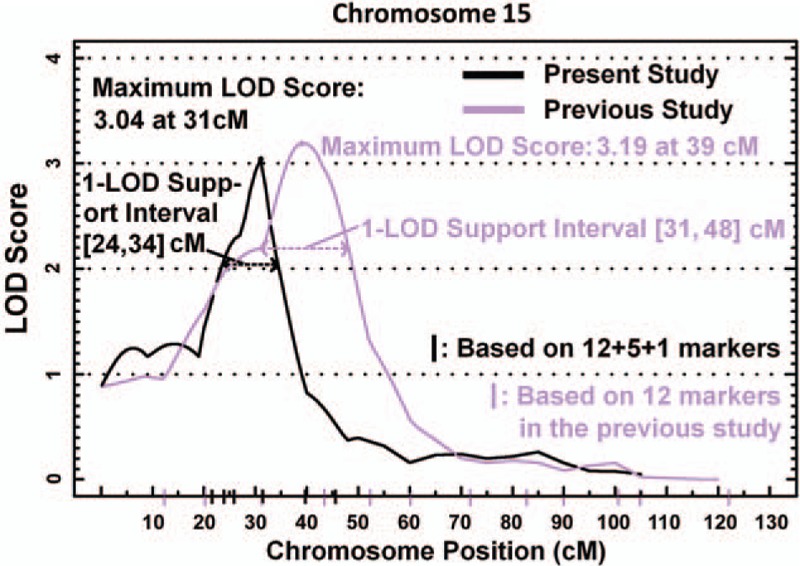
Genetic association of *RYR3* with circulating adiponectin levels. Genome-wide multipoint linkage analysis of plasma adiponectin levels. Multipoint LOD scores using are depicted by solid lines (blue line indicates previous LOD scores using 12 microsatellite markers; black line indicates LOD scores using additional 6 microsatellite markers). LOD = logarithm of odds, *RYR3* = ryanodine receptor 3.

In a previous genome-wide linkage scan in the SAPPHIRe Chinese family cohort, we identified a linkage signal on chromosome 15 at 39 cM (LOD score = 3.19 with 1-LOD support interval at 31–48 cM) with plasma adiponectin levels using microsatellite marker spanned at an interval of approximately 17 cM. To refine this signal, we placed additional 6 microsatellite markers and mapped a quantitative trail locus (QTL) located at 31 cM (LOD = 3.04 with 1-LOD support interval at 24–34 cM) (Fig. 1). Compared to original linkage signal, the additional markers resulted in a peak shifted by about 8 cM and the 1-LOD support interval shortened by 7 cM. Within this region, we further genotyped a total of 68 SNPs for the 12 genes including the *CHRNA7*, *ARHGAP11A*, *SCG5*, *GREM1*, *RYR3*, *CHRM5*, *SLC12A6*, *NUT*, *AYTL3*, *GOLGA8A*, *AQR*, and *RASGRP1* genes.

**Figure 2:**
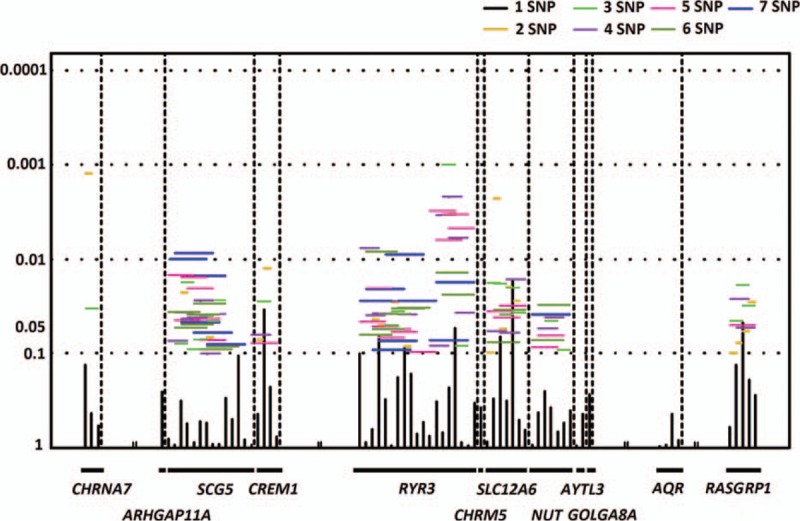
Regional fine mapping of the linkage signal using 68 SNPs in 12 genes. Sliding window analysis for haplotype association with plasma adiponectin is shown. The global *P* values (shown as *Y*-axis) for single SNP or 2-, 3-, 4-, 5-, 6-, and 7-SNP haplotypes are shown as horizontal bar. Single-locus *P* values are shown as vertical bar. SNP = single-nucleotide polymorphism.

**Figure 3:**
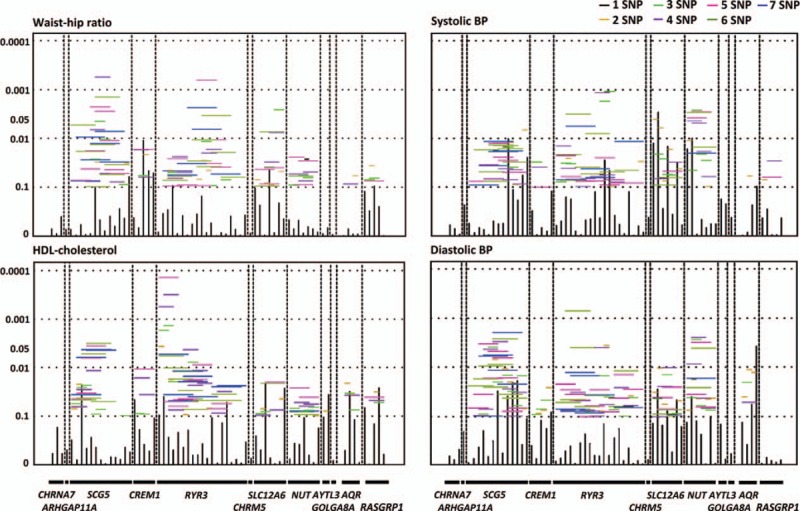
Sliding window analysis for haplotype association with waist–hip ratio, high-density lipoprotein (HDL) cholesterol, and systolic and diastolic blood pressure (BP) is shown. The global *P* values (shown as *Y*-axis) for single SNP or 2-, 3-, 4-, 5-, 6-, and 7-SNP haplotypes are shown. Single-locus *P* values are shown as vertical bar. SNP = single-nucleotide polymorphism.

Sliding window analysis using family-based association test found that haplotypes of the *RYR3* gene had strongest association with plasma adiponectin levels (*P* = 0.001) (Fig. 2). *RYR3* haplotypes were also associated with systolic (*P* = 0.001) and diastolic (*P* = 7.1 × 10^−4^) blood pressure and HDL-C (*P* = 1.4 × 10^−4^) (Fig. 3).

### 3.2 Correlation between *RYR3* and *adiponectin* expression in human adipose tissue

**Figure 4:**
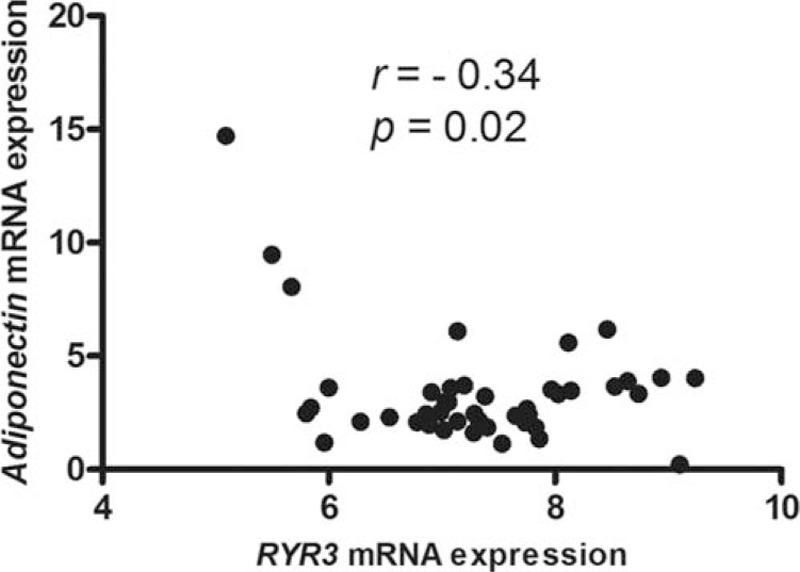
Association between *RYR3* and *adiponectin* expression in human (A) omental and (B) subcutaneous adipose tissues. *RYR3* = ryanodine receptor 3.

Since adiponectin is expressed in adipose tissues, we examined the correlation between expression of *RYR3* and *adiponectin* in 44 human adipose tissues. We found an inverse relationship between *RYR3* and *adiponectin* expression in omental (*r* = −0.34, *P* = 0.02) adipose tissues (Fig. 4), suggesting that *RYR3* is a negative regulator of adiponectin; this finding is consistent with a previous report that silencing of *RYR3* both in vitro and in vitro elevated plasma insulin and improved insulin sensitivity.^[17]^

## 4 Discussion

In this study, we refined the genome-wide linkage signal of our previous study using additional microsatellite marker and mapped a QTL located in chromosome 15 at 31 cM with LOD of 3.04. Within this linkage region, we identified variants in the *RYR3* gene associated with plasma adiponectin. *RYR3* genetic variants were also associated with HDL-C, waist–hip ratio, and blood pressures. In addition, *RYR3* expression was inversely associated with *adiponectin* expression in human abdominal adipose tissues.

Adiponectin is an adipokine with potent insulin-sensitizing and antiatherosclerosis actions. Previous genome-wide association studies (GWAS) or meta-analyses of GWAS in European population have identified ∼10 SNPs associated with plasma adiponectin levels.^[6,7,10,11]^ The SNPs are also associated with altered risk of type 2 diabetes, plasma triglycerides, HDL-C, blood pressure, and waist–hip ratio.^[6,7,10,11]^

Here we reported that novel variants in the *RYR3* gene are associated with plasma adiponectin levels in Chinese population. Interestingly, a recent European GWAS also identified a variant in the *RYR3* gene (rs6495001) associated with plasma adiponectin in 2097 men.^[10]^ This variant is associated with plasma adiponectin level in both stage 1 (*P* = 8.8 × 10^−6^) and stage 1 + 2 (*P* = 0.039) analyses.^[10]^ We tried to replicate this finding in our dataset. However, this SNP is not polymorphic in Chinese population (data not shown) and therefore a direct comparison is not possible. However, the appearance of similar signals from 2 independent studies suggests *RYR3* as a genetic determinant of plasma adiponectin.

The *RYR3* gene encodes for a cation channel that releases intracellular calcium storage pools. Three RyR isoforms were identified with tissue-specific expression pattern including RyR1, RyR2, and RyR3. RyR1 is expressed mainly in skeletal muscle while RyR2 is expressed in heart. RyR3 is ubiquitously expressed. Knockdown of RyR3 in preadipocytes has been shown to upregulate adiponectin promoter activity, enhance adiponectin expression, and cause more adiponectin secretion into the medium through activation of activating transcription factor 3.^[18]^ Activating transcription factor 3 is an inducible transcriptional repressor controlling adiponectin expression.^[18,19]^ Silencing of RyR3 with small interfering RNA in *db*/*db* mice and high-fat diet-induced obese mice also increased serum adiponectin level and improved insulin sensitivity.^[17]^ Consistently, we observed an inverse relationship between *RYR3* and *adiponectin* expression in human adipose tissue. Collectively, these data suggest that RyR3 is a suppressor of adiponectin expression.

Our data have some unique strengths. First, this is the first genome-wide linkage analysis and fine association mapping for plasma adiponectin in Chinese population. Second, the family-based design of this study prevented potential confounding by population stratification, a frequent cause for spurious association in case–control association studies.^[9]^ Third, the metabolic phenotypes of the SAPPHIRe cohort are complete, making a comprehensive testing for metabolic phenotypes feasible. However, there are some limitations of our study. First, the significance level of most single-locus association did not pass the study-wide significance threshold. Based on the linkage method, a LOD score exceeding 3.3 was considered to be of genome-wide significance for evidence of linkage, whereas a LOD score >1.9 was suggestive of evidence for linkage. However, a LOD of 3.04 of our studies can still be considered as a highly suggestive signal almost reaching genome-wide significance. Second, although the association of *RYR3* genetic variation with plasma adiponectin was also observed in another European GWAS in men,^[10]^ further replication is still needed to confirm the association. Third, only 68 SNPs were used for association testing in our study across a large chromosomal region. A finer mapping would help to identify variants with best association within this region.

## 5 Conclusions

In summary, we demonstrated for the first time that genetic variants in the *RYR3* gene are associated with plasma adiponectin in Chinese, which is consistent with the finding of a recent GWAS. ^[10]^ A reverse relationship between *RYR3* and *adiponectin* expression in human adipose tissue was also observed. These data, together with previous functional investigations, support a role of *RYR3* in the regulation of adiponectin expression.
